# Graphene/h-BN Heterostructures for Vertical Architecture of RRAM Design

**DOI:** 10.1038/s41598-017-08939-2

**Published:** 2017-08-29

**Authors:** Yi-Jen Huang, Si-Chen Lee

**Affiliations:** Graduate Institute of Electronics Engineering, National Taiwan University, Taipei, Taiwan

## Abstract

The development of RRAM is one of the mainstreams for next generation non-volatile memories to replace the conventional charge-based flash memory. More importantly, the simpler structure of RRAM makes it feasible to be integrated into a passive crossbar array for high-density memory applications. By stacking up the crossbar arrays, the ultra-high density of 3D horizontal RRAM (3D-HRAM) can be realized. However, 3D-HRAM requires critical lithography and other process for every stacked layer, and this fabrication cost overhead increases linearly with the number of stacks. Here, it is demonstrated that the 2D material-based vertical RRAM structure composed of graphene plane electrode/multilayer h-BN insulating dielectric stacked layers, AlO_x_/TiO_x_ resistive switching layer and ITO pillar electrode exhibits reliable device performance including forming-free, low power consumption (P_set_ = ~2 μW and P_reset_ = ~0.2 μW), and large memory window (>300). The scanning transmission electron microscopy indicates that the thickness of multilayer h-BN is around 2 nm. Due to the ultrathin-insulating dielectric and naturally high thermal conductivity characteristics of h-BN, the vertical structure combining the graphene plane electrode with multilayer h-BN insulating dielectric can pave the way toward a new area of ultra high-density memory integration in the future.

## Introduction

Recently, with the increasing demand for improved computing performance of mobile electronic products, the need for storage electronic device like flash memory as one of the vital components has also climbed up accordingly. However, as the integration density continues rising, flash memory is reaching the physical limitation due to the reliability issues as well as process cost^1–3^. Although, the current flash technology also has found a way to overcome the scaling limit by adopting three dimensional (3D) architecture to achieve high density, the endurance and large power consumption are still fundamental issues that need to be surmounted^4, 5^. Thus various emerging memories with new storage mechanisms have been put forward as candidates for flash memory replacement, such as ferroelectric random access memory (FRAM)^6–8^, magnetoresistive RAM (MRAM)^9–11^ and phase-change RAM (PRAM)^12–15^, etc. Among them, the resistive random access memory (RRAM) is the most promising candidate to overcome the technological limitations for the next-generation non-volatile memory, because of its advantages, such as high-speed operation, low power consumption, high endurance, and long retention time^16–26^. The simple fabrication process, good scaling capability and low bit cost^27–29^ are some of the potential merits presented in some recent studies to demonstrate that several 3D vertical RRAM are competitive with the high bit density of 3D NAND flash memory. However, the sneak current through neighboring cells is still a problem in the 3D crossbar arrays structure. Thus, non-linear selector devices are needed in the circuit to suppress the sneak current paths^30–33^. In addition, a few studies mainly focus on the stacking potential of the device in a 3D architecture by introducing the carbon-based electrodes, including carbon nanotube (CNT) and graphene to attain the ultra-high storage density and realize the low power consumption properties^34, 35^. Nonetheless, the large operation current and small memory window still have room for improvement. Besides, the thermal crosstalk is also critical issue that should be considered in the 3D architecture RRAM applications. The Joule heating inside the RRAM device not only impacts on the reliability of the device, but also degrades the neighboring devices during the programming^36^. The carbon-based electrodes have the high thermal conductivity as CNT and graphene that can be used to enhance lateral heat spreading^34, 35^. However, the ability of heat spreading in insulating dielectrics between these different layers of electrodes is still not good enough.

Fortunately, hexagonal boron nitride (h-BN) has been suggested as an ideal insulating dielectric layer for Two-dimensional (2D) electronic devices owing to its excellent properties such as wide direct bandgap (5.9–6.1 eV), high mechanical hardness and resilience. Because of a small lattice mismatch (1.7%) with graphene and atomically smooth surface of h-BN, the electron mobility of graphene electronic devices found on h-BN is remarkably higher than that found on silicon dioxide^37^. For graphene-based interconnect applications, the impact of molecular adsorbates and oxidation can be subdued by encapsulating the graphene with a top h-BN films which are chemically inert^38^. In addition, it has been reported that the basal plane thermal conductivity of bulk sample can be as high as 390 W m^−1^ K^−1^ at room temperature^37^. This value not only almost matches that of copper and silver but also is higher than that for the SiO_2_ dielectric used in current-generation silicon electronic devices. Although the thermal conductivity along the c-axis of h-BN is as low as 2 W m^−1^ K^−1^ because of anisotropic layered crystal structure, the high basal plane thermal conductivity can be used as a functional gate dielectric of 2D electronic devices for thermal management of the channel heat^37^.

As mentioned above, in order to satisfy the exponentially grown needs for big-data storage, to increase the memory storage capability including the low operation current and large memory window, and to reduce the influence of thermal crosstalk, in this paper, a vertical architecture of RRAM was proposed and successfully demonstrated by 2D materials of graphene (plane electrode)/multilayer h-BN (insulating dielectric) stacked layers, with the aluminum oxide (AlO_x_, x = 1.23) and titanium oxide (TiO_x_, x = 1.76) resistive switching layers and indium tin oxide (ITO) pillar electrode. By combining with multilayer h-BN insulating dielectric and graphene plane electrode, the vertical structure of RRAM not only exhibits reliable device performance and low power consumption, but also shows huge potential for ultra high-density memory integration and per-bit lithography cost reduction by increasing the stacked layers in the future.

## Results

### Structure and fabrication process of 2D material-based vertical RRAM

**Figure 1:**
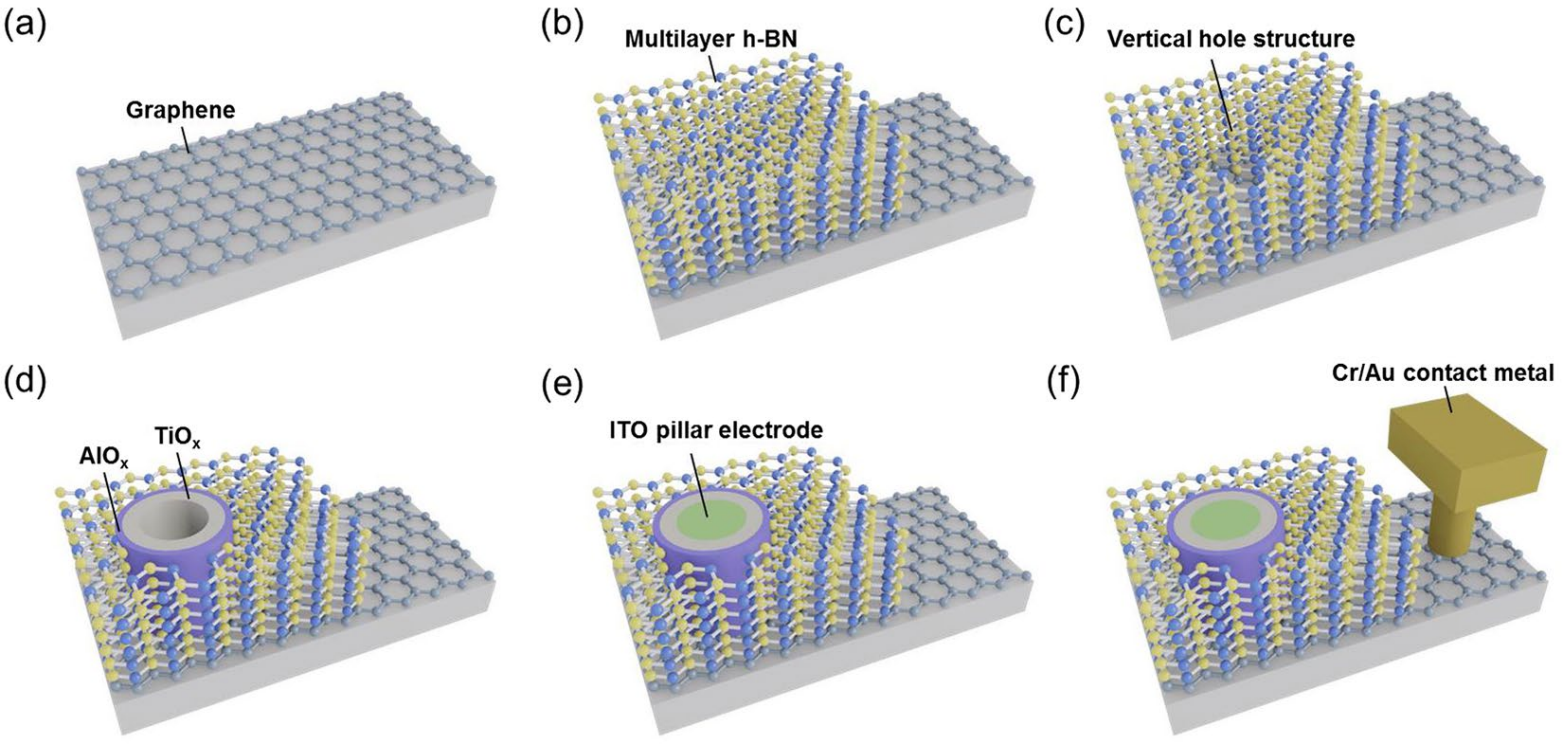
The fabrication flows of a single 2D material-based vertical RRAM. (**a**) Wet transfer the graphene plane electrode on SiO_2_ (90 nm) substrate. (**b**) Wet transfer the hexagonal boron nitride (h-BN) insulating dielectric on graphene/SiO_2_ (90 nm) substrate. After pattering the hole structure by EBL technique, **(c)** the vertical hole structure with the diameter of 80 nm is etched by dry etching technique. (**d**) Deposition of AlO_x_ (1 nm) and TiO_x_ (10 nm) resistive switching layer by ALD technique, respectively. (**e**) Deposition of ITO (50 nm) pillar electrode by sputtering technique. (**f**) Deposition of Cr (5 nm) and Au (100 nm) on the graphene plane electrode for contact metal by thermal evaporation technique, respectively.

Figure 1(a) to (f) illustrate the schematic fabrication processes of 2D material-based vertical RRAM device. In short, a layer of poly (methyl methacrylate) (PMMA) is first coated on the chemical vapor deposition (CVD) single/double layer graphene/Cu foil, which is then transferred onto the SiO_2_ substrate via a wet transfer methods^39^, as shown in Fig. 1(a). Subsequently, another layer of PMMA is coated on the CVD multilayer h-BN/Cu foil. Then, the multilayer h-BN is transferred onto the graphene/SiO_2_ substrate via a same transfer method, as shown in Fig. 1(b). A vertical hole structure is then patterned by electron beam lithography and then etched by dry etching, as shown in Fig. 1(c). Next, the AlO_x_/TiO_x_ resistive switching layer and ITO pillar electrode are realized via atomic layer deposition (ALD) and sputtering processes, respectively, as shown in Fig. 1(d and e). Finally, a Cr/Au film is deposited onto the graphene plane electrode for contact metal, as shown in Fig. 1(f) (Detailed Device fabrication processes are described in Methods section).

**Figure 2:**
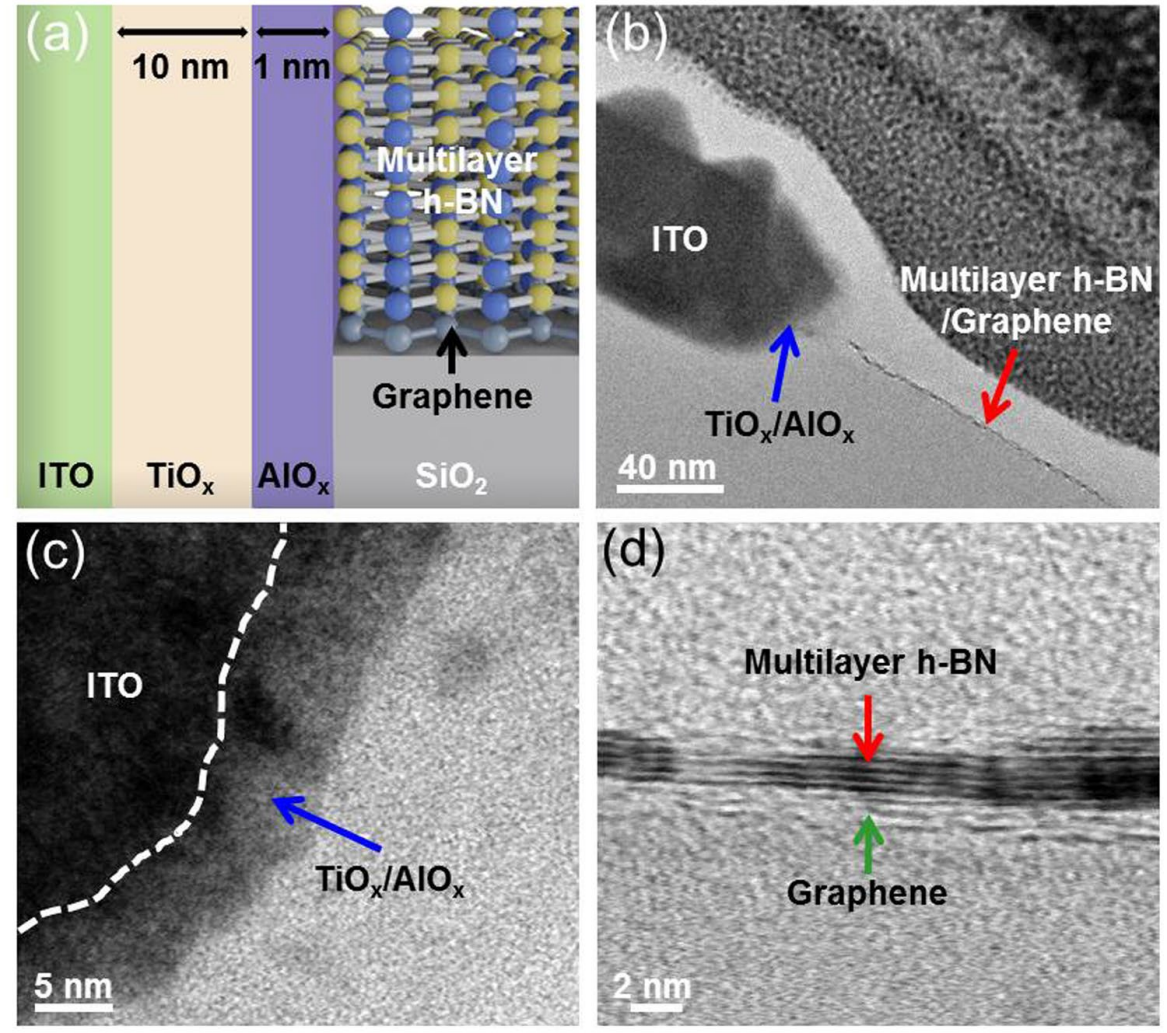
Structure of 2D material-based vertical RRAM. (**a**) A schematic cross-sectional of the 2D material-based vertical RRAM. (**b**) Cross-sectional bright-field transmission electron microscopy (TEM) image of the as-prepared RRAM cell in this study. (**c**) Cross-sectional bright field scanning transmission electron microscopy (STEM) image of the AlO_x_/TiO_x_ resistive switching layers and (**d**) the graphene (plane electrode)/multilayer h-BN stacked layers of the 2D material-based vertical RRAM.

Figure 2(a and b) show a cross-sectional view and bright field transmission electron microscope (TEM) image of the 2D material-based vertical RRAM structure. From the TEM image in Fig. 2(b), the AlO_x_/TiO_x_ resistive switching layer (blue arrow) is located along the sidewall between ITO pillar electrode and graphene plane electrode vertically. Besides, the multilayer h-BN completely covers on the graphene plane electrode (red arrow), which not only protects the graphene but also suppresses the leakage current from dielectric layer. To further confirm the structures of resistive switching layers and graphene (plane electrode)/multilayer h-BN stacked layer, Fig. 2(c and d) reveal the cross-sectional bright field scanning transmission electron microscopy (STEM) images of the AlO_x_/TiO_x_ resistive switching layer and the graphene/multilayer h-BN stacked layers, respectively. The resistive switching layer with the thickness about 11 nm is uniformly coated on the sidewall of the vertical hole structure. In addition, the multilayer h-BN with the thickness about 2 nm completely covers on the single/double graphene layer. Because of the ultrathin-insulating dielectric layer and naturally high thermal conductivity characteristics of h-BN, this vertical structure combining the graphene (plane electrode) and multilayer h-BN (insulating dielectric) can exhibit huge potential for future ultra high-density memory integration and per-bit lithography cost reduction by increasing the stacked layers.

### Electrical performance of 2D material-based vertical RRAM

**Figure 3:**
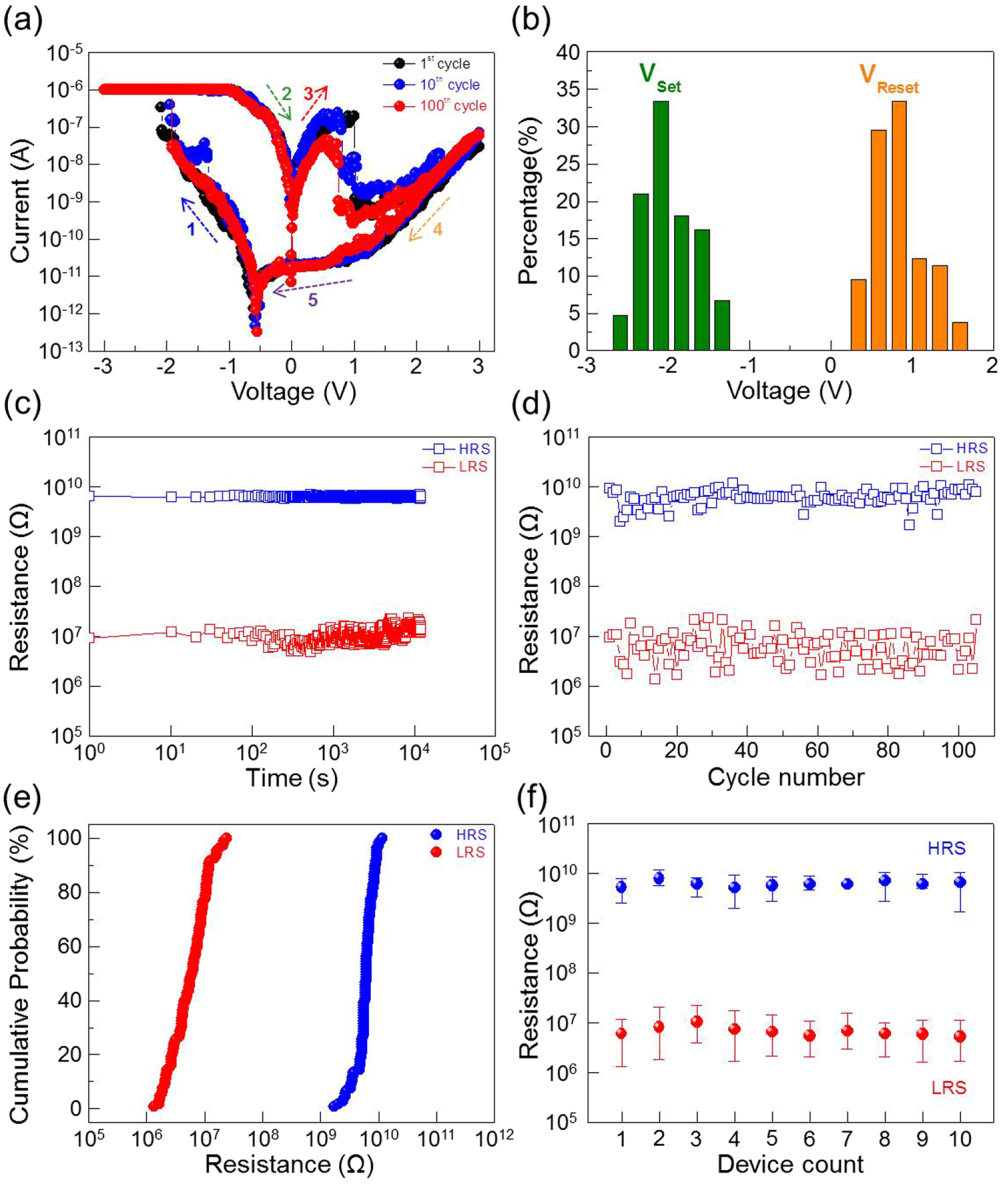
Electrical performance of 2D material-based vertical RRAM. (**a**) Current-voltage diagrams of the first, 10^th^, and 100^th^ bipolar switching cycles of the device. (**b**) Distribution of the setting and resetting voltages in 100 successive cycles in (**a**). (**c**) Plots of the resistances of the device at HRS and LRS at room temperature in the data retention test. (**d**) Plots of the resistances of the device at HRS and LRS versus cycle number in the endurance test. (**e**) Cumulative probability of the resistances in (**d**). **(f)** Device-to-device tests of HRS and LRS for ten successive cycles. The voltage for reading the resistance in (**c**–**f**) is −0.1 V.

**Figure 4:**
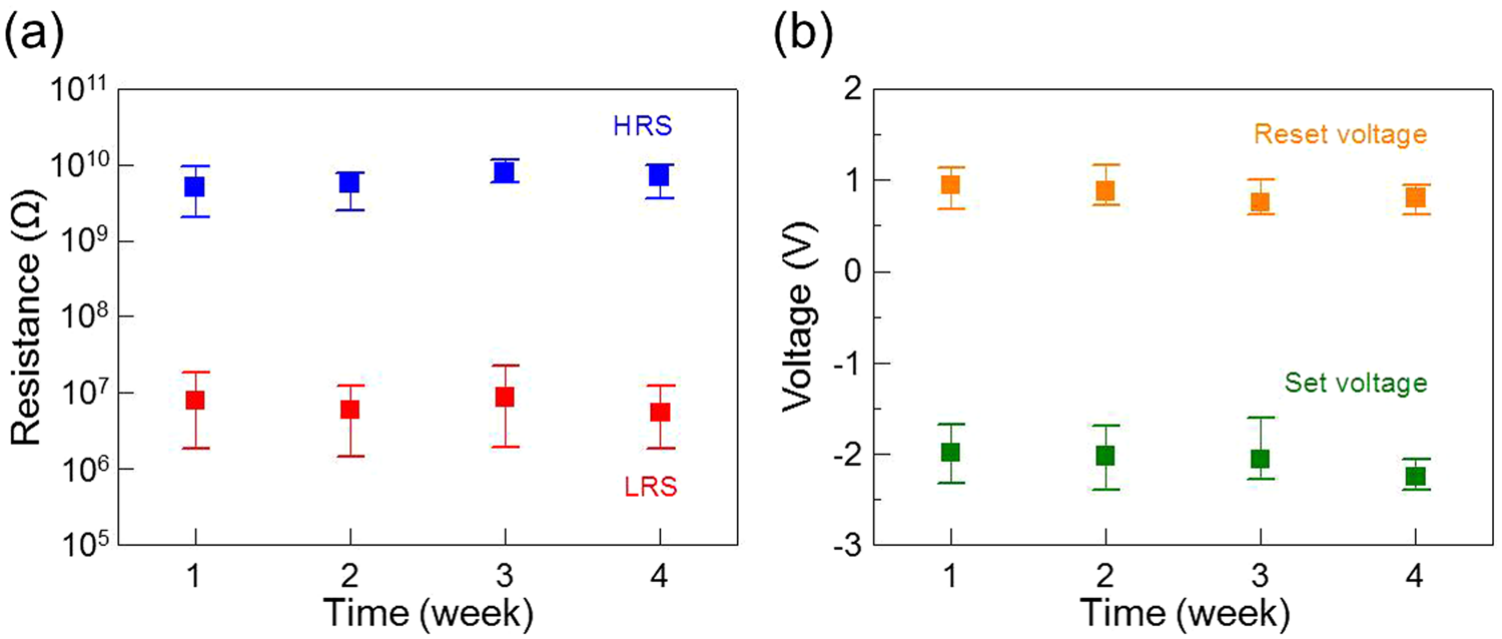
Time-dependent characteristics of 2D material-based vertical RRAM. (**a**) Distribution of HRS and LRS; (**b**) The setting and resetting voltages of RRAM for ten random cells during the four weeks.

Figure 3(a) shows the typical bipolar current-voltage (I-V) characteristics of 2D material-based vertical RRAM device in this study. For the electrical measurements, a DC voltage is applied to the ITO pillar electrode and the graphene plane electrode is grounded. Typically, a “forming” step is required to create conducting filaments in order to operate the RRAM devices^19, 20, 22–24^. In the forming process, a voltage higher than the RRAM operation voltage is applied, so that the device performance may sometimes be degraded due to the induced high current in this step. For this study, the as-prepared device is at the high resistance state (HRS), but the initial high-voltage forming step is not required for the following memory operations. During the voltage sweep from 0 to −3 V, the device is switched to the low resistance state (LRS) at the setting voltage (<−3 V). In the reversed sweep to 3 V, the device is switched back to the HRS at the resetting voltage (<2 V). A compliance current (I_cc_) of 1 μA is applied during the measurements, so that, after setting the device to LRS, the current will not be so large to cause irreversible device breakdown. This resistive switching characteristic sustains after 100 switching cycles. The setting and resetting voltages were counted from the endurance test. The distributions of the setting and resetting voltages are shown in Fig. 3(b); V_set_ is −2.03 ± 0.32 V (mean value: −2.03 V; standard devivation: 0.32 V), and V_reset_ is 0.87 ± 0.32 V (mean value: 0.87 V; standard devivation: 0.32 V). The average power consumption in the setting process (P_set_ = V_set_ × I_cc_) is ~2 μW, and that in the resetting process (P_reset_ = V_reset_ × I_reset_) is ~0.2 μW. Such low power consumption suggests that the device is very suitable for memory applications in the future. In addition, the device has excellent data retention as shown in Fig. 3(c). The resistances at HRS and LRS, read at −0.1 V at room temperature, are very stable after 10^4^ s. The device is therefore nonvolatile, and the reading operation is nondestructive. Figure 3(d) shows the endurance of the device; the resistances of the LRS and HRS (measured at −0.1 V) are consistently and the on/off ratio is larger than 300 after to 100 sweeping cycles. The resistances of the device at HRS and LRS are also uniform, as shown in Fig. 3(e), with mean values of 6.3 GΩ and 7.2 MΩ, respectively. Because the memory window of the resistance states directly affects the sensing margin of memory devices, large memory window is required for reliable reading operation. Moreover, the large memory window can realize multi-level cell (MLC) applications^40^. The device-to-device uniformity of 2D material-based vertical RRAM devices are investigated, in terms of the cumulative distributions of resistance values for ten random memory devices under ten continuous switching cycles. Figure 3(f) shows the statistical data for the HRS and LRS resistances obtained by a −0.1 V read voltage. It can be seen from Fig. 3(f) that both HRS and LRS exhibit concentrated distributions and a large memory window between the two states can be observed. Time-dependent reliability tests of 2D material-based vertical RRAM devices have been demonstrated from ten random memory devices during the four weeks. The distributions of resistance state and operation voltage still maintain a good uniformity and reliability, as shown respectively in Fig. 4(a and b). These results mean that the impact of molecular adsorbates and oxidation can be suppressed by covering the graphene plane electrode with a h-BN insulating dielectric layer which has the chemical inertness property.

### Electrical properties of 2D material-based vertical RRAM

**Figure 5:**
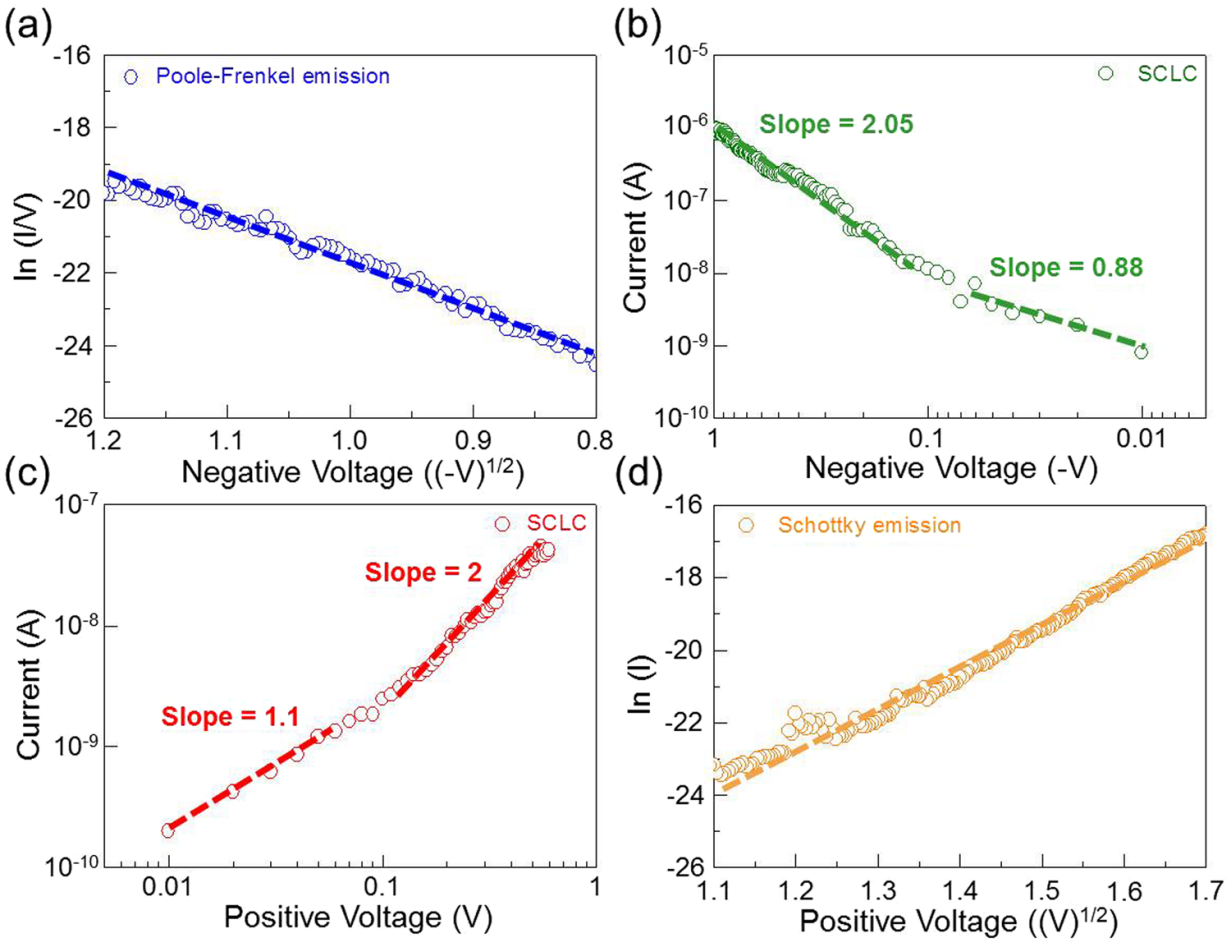
Electrical characteristics of 2D material-based vertical RRAM. The current conduction mechanism in the setting process: (**a**) the ln (I/V) versus V^1/2^ relationship of the device in HRS is fitted to Poole-Frenkel emission model and (**b**) double-logarithmic plots of the current-voltage relationship of the device in LRS. In the resetting process: (**c**) double-logarithmic plots of the current-voltage relationship of the device in LRS and (**d**) the ln (I) versus V^1/2^ relationship of the device in HRS is fitted to Schottky emission model.

In order to better understand the current conduction mechanism of 2D material-based vertical RRAM device, I-V curves are fitted with theories related to different conduction mechanisms for each resistance state in negative voltage (as labeled by blue arrow-1 and green arrow-2 in Fig. 3(a)), positive voltage (as labeled by red arrow-3 and orange arrow-4 in Fig. 3(a)), and the voltage transition (as labeled by purple arrow-5 in Fig. 3(a)) regions. In the setting process (negative voltage region), as labeled by blue arrow-1 in Fig. 3(a), the current increases with the increase of negative voltage from 0.6 V to V_set_, as labeled by blue arrow-1 in Fig. 3(a), is replotted in Fig. 5(a) as ln (I/V) versus V^1/2^. The linear dependence indicates that the current conduction mechanism is governed by the Poole-Frenkel (P-F) emission^41^. The P-F emission mechanism is a bulk effect coming from the lowering of the Coulomb potential barrier of trap sites by an external electric field. After the setting process of the device at LRS, as labeled by green arrow-2 in Fig. 3(a), the I-V curve is replotted in double-logarithmic scale in Fig. 5(b), a tiny conducting filament is formed. Thus, the current varies linearly with voltage (I ∝ V) in the small bias region, followed by the quadratic dependence (I ∝ V^2^) in the larger bias region. Such I-V relation is in agreement with the characteristics of the trap-controlled space-charge-limited current (SCLC)^42–45^, and the dominating trap sites in this device are the oxygen vacancies. A similar conducting mechanism is also noted in the resetting process, as shown in Fig. 5(c) (as labeled by red arrow-3 in Fig. 3(a)). The trap-controlled space-charge-limited current again dominates the current conduction mechanism of LRS^42–45^. After the resetting process of the device at HRS, as labeled by orange arrow-4 in Fig. 3(a), the I-V curve is replotted in Fig. 5(d) as ln (I) versus V^1/2^, the current conduction mechanism in HRS can be well fitted by the Schottky emission theory^41^. The leakage current of HRS is due to thermal emission of electrons over the effective Schottky barrier at graphene/AlO_x_ interface. It is worth noting that in the voltage transition region, as labeled by purple arrow-5 in Fig. 3(a), the current decreases gradually with the change of bias from 1.2 to −0.6 V, which behavior like the reverse-biased current of the effective Schottky barrier finally balanced by the forward-biased current to give a net zero current at −0.6 V.

## Discussion

**Figure 6:**
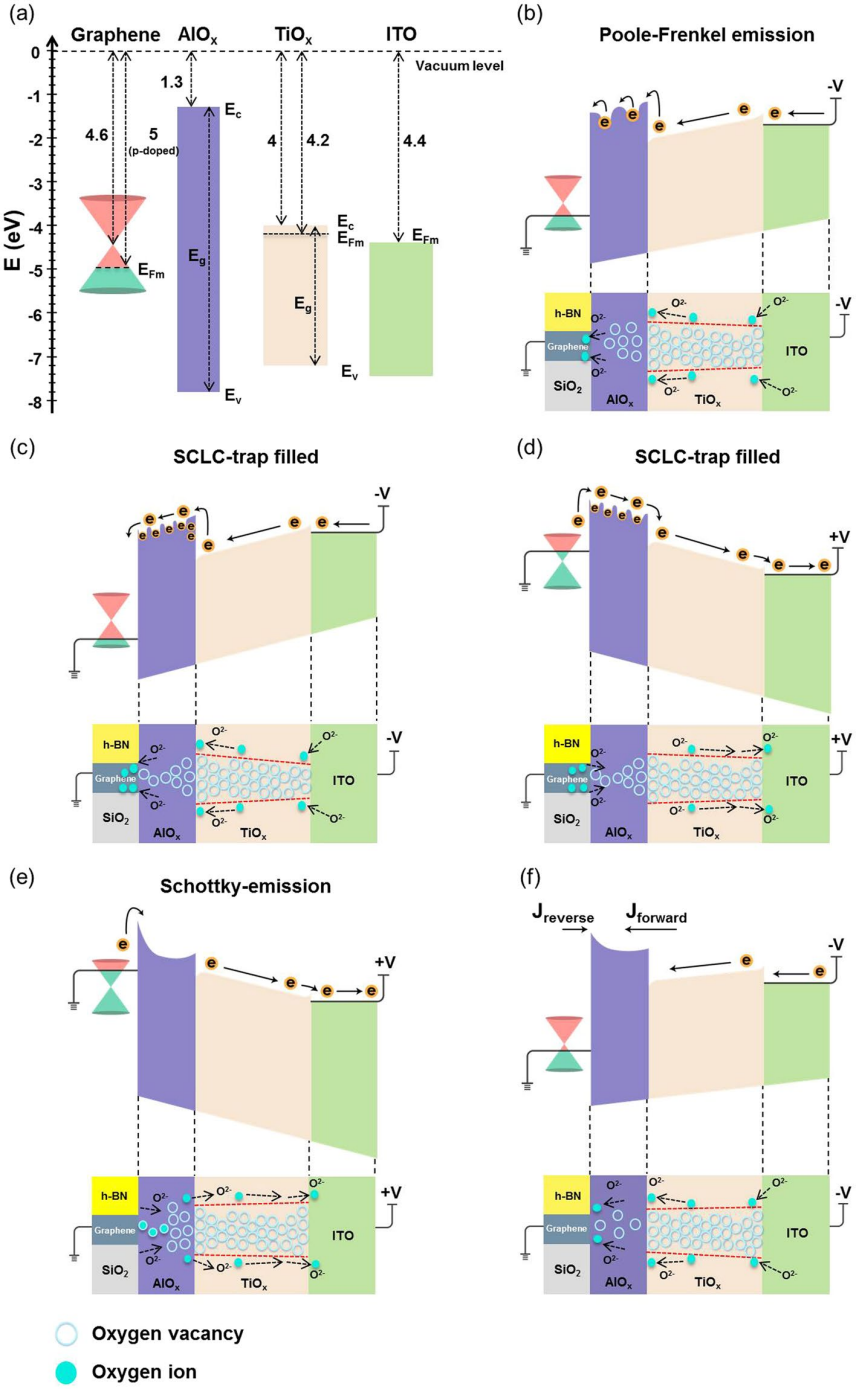
Schematic illustration of the resistive switching mechanism of 2D material-based vertical RRAM. (**a**) Schematic view of electronic band diagram of graphene/AlO_x_/TiO_x_/ITO system; all values are in eV. (**b**–**f**) Schematic illustrations of the variability of electronic band diagram and migration of the oxygen ions due to the applied voltage. After device preparation, the concentration of oxygen vacancies (cyan circles) at the initial resistance state (IRS) is low in the AlO_x_ layer and high in the TiO_x_ layer. When the applied negative voltage is larger, oxygen ions (aquamarine circles) are pushed (indicated by black arrows) into the TiO_x_ layer and recombine with the oxygen vacancies (**b**). Thus, the filament (the red dashed region) in the TiO_x_ layer shrinks. In the AlO_x_ layer, oxygen ions are pulled (indicated by black arrows) to the graphene plane electrode. At the same time, the current conduction mechanism is in good agreement with Poole-Frenkel emission model (**b**). When the applied voltage reaches the V_set_, the tiny filaments are formed in AlO_x_ layers, and the device is switched to the low-resistance state (LRS). At this time, the current conduction mechanism is changed from Poole-Frenkel emission to trap-controlled SCLC with the trap filled property (**c**). During the resetting process with the applied positive voltage smaller than the V_reset_, oxygen ions in the TiO_x_ layer are pulled out (indicated by black arrows) to the ITO pillar electrode. Meanwhile, some oxygen ions are pushed (indicated by black arrows) from the graphene plane electrode to the AlO_x_ layer. The current conduction mechanism is still the trap-controlled SCLC with the trap filled property (d). When the applied voltage reaches the V_reset_, recombination of oxygen ions with the vacancies in the AlO_x_ layer causes dissolution of the tiny filaments (**e**). The sample is at the high-resistance state (HRS). At last, the current conduction mechanism is changed from trap-controlled SCLC to Schottky emission model (**e**). In the voltage transition region, the reverse bias injection current at the graphene/AlO_x_ Schottky barrier from the graphene side begins to be compensated by the forward injection current from the TiO_x_ side (**f**).

To further elaborate the aforementioned conduction mechanisms involved, the corresponding carrier transport mechanisms and the formation of conducting filaments based on the push-pull mechanism are presented in a diagrammatic manner in order to explain the resistive switching characteristics^25, 26^, as shown in Fig. 6. Schematic view of electronic band diagram of the 2D material-based vertical RRAM with graphene/AlO_x_/TiO_x_/ITO structure is shown in Fig. 6(a). Here, the assumed wok function value of graphene^46^, TiO_x_^47^, and ITO is 5 eV, 4.2 eV and 4.4 eV, respectively; the electron affinity of AlO_x_^48^ and TiO_x_^47^ is 1.3 eV and 4 eV; E_g_ is the bandgaps (6.4 eV for AlO_x_^48^ and 3.4 eV for TiO_x_^47^); E_c_ and E_v_ are the conduction band and valence band edges. It is worth noting that graphene plane electrode becomes p-doped by the H_2_O dopant introduced during the wet transfer process^49^, indicating that the work function of graphene is changed from the charge-neutral point 4.6 eV to 5 eV. In this study, TiO_x_ deposited by ALD is usually oxygen deficient^25, 26^, so that the conducting filaments have formed in the TiO_x_ layer. The AlO_x_ layer has a better insulating property; nonetheless, it is so thin that the electroforming step is not required.

During the setting process, a negative voltage is applied to the ITO pillar electrode and starts to push oxygen ions into the TiO_x_ layer from ITO pillar electrode; meanwhile, the oxygen ions start to be pulled from the AlO_x_ barrier layer to the graphene plane electrode. The current conduction is limited by the AlO_x_ barrier layer. When the applied negative voltage becomes larger (as labeled by blue arrow-1 in Fig. 3(a)), the oxygen ions migration is enhanced and the continuous pull of the oxygen ions cause the increase of the oxygen vacancies inside the AlO_x_ layer. Therefore, the current conduction mechanism at HRS shows the P-F emission property across the AlO_x_ barrier layer^41^, as shown in Fig. 6(b). After the setting process, the device is at LRS (as labeled by green arrow-2 in Fig. 3(a)), and the number of filaments in the TiO_x_ layer is reduced due to the push process of oxygen ions from ITO pillar electrode to TiO_x_ layer which filled up oxygen vacancies, but a few conducting filaments are formed in the AlO_x_ layer, and the current flow exhibits the linear and then trap-controlled SCLC phenomenon^42–45^ at lower negative voltage (as labeled by green arrow-2 in Fig. 3(a)), as shown in Fig. 6(c). Thus, the power consumption in this RRAM device is low. In addition, the graphene plane electrode can be used as oxygen capturing layer^34^. The oxygen ions that pull from AlO_x_ layer can be highly mobile in graphene and may form a covalence bond with the broken bonds of graphene after the setting process, and the process is reversed during the resetting process.

During the resetting process at LRS (as labeled by red arrow-3 in Fig. 3(a)), the current conduction mechanism is shown in Fig. 6(d), the positive voltage sweep pulls the oxygen ions from the TiO_x_ layer to the ITO pillar electrode, and pushes the oxygen ions from graphene plane electrode into the AlO_x_ layer. Before rupturing the few conducting filaments inside the AlO_x_ layer, the current flow still exhibits the linear and then trap-controlled SCLC phenomenon^42–45^. When the applied voltage is larger than V_reset_ (as labeled by orange arrow-4 in Fig. 3(a)), the continuous pull process of oxygen ions will increase the number of filaments inside the TiO_x_ layer, but the push of the oxygen ions from the graphene plane electrode to the AlO_x_ layer will rupture the few conducting filaments near the interface, and results in the Schottky emission property of the current flow^41^, as shown in Fig. 6(e). At this time, the current flow of HRS is due to thermal emission of electrons over the effective Schottky barrier between the graphene plane electrode and the AlO_x_ layer^41, 50^. It is worth noting that in the voltage transition region (as labeled by purple arrow-5 in Fig. 3(a)), the current conduction mechanism is shown in Fig. 6(f), when the voltage is changed from 1.2 to −0.6 V, the reverse bias injection current at the graphene/AlO_x_ Schottky barrier from the graphene side begins to be compensated by the forward injection current from the TiO_x_ side. Eventually, both injection currents are balanced at a voltage of −0.6 V, leading to a net zero current. The current conduction mechanism is shown in Fig. 6(f), when the negative voltage exceeds −0.6 V, the injection current from the TiO_x_ side dominates and the bottle neck for current conduction shifts to the AlO_x_ barrier layer, as shown in Fig. 6(b).

In summary, 2D material-based vertical RRAM structure composed of graphene plane electrode/multilayer h-BN insulating dielectric stacked layers, AlO_x_/TiO_x_ resistive switching layer and ITO pillar electrode exhibits reliable device performance with low power consumption. Because of the ultrathin-insulating dielectric layer (~2 nm) and naturally high thermal conductivity characteristics of h-BN, the proposed vertical RRAM exhibits huge potential for future ultra high-density memory integration and per-bit lithography cost reduction by increasing the stacked layers. This RRAM device is forming-free for resistive switching, with uniformity setting and resetting voltages. It also has uniform resistances at LRS and HRS and a large memory window (>300). It is worth noting that the average power consumption in the setting process is ~2 μW, and in the resetting process is ~0.2 μW.

## Methods

### Device fabrication

Detailed fabrication processes of the 2D material-based vertical RRAM structure is illustrated as follows: At first, a layer of poly(methyl methacrylate) (PMMA) was coated on the chemical vapor deposited single/double layer graphene/Cu foil and multilayer h-BN/Cu foil which were purchased from graphene supermarket, and then, the graphene and h-BN films were transferred onto the SiO_2_ (90 nm) substrate via a wet transfer approach^39^, respectively, to form the graphene plane electrode (first transfer layer)/multilayer h-BN insulating dielectric (second transfer layer) stacked layers. The optical microscopy and Raman spectroscopy images of the graphene/multilayer h-BN stacked layers after the wet transfer processes were shown in Supplementary Fig. S1. Subsequently, the vertical hole structures with the diameter of 80 μm were fabricated by electron beam lithography (EBL) with PMMA bi-layer resist and dry etching (Supplementary Fig. S2). Next, 1 nm aluminum oxide (AlO_x_, x = 1.23) and 10 nm titanium oxide (TiO_x_, x = 1.76) layers were well deposited sequentially at 200 °C by using the atomic layer deposition (ALD) process. Trimethylaluminum (Al(CH_3_)_3_, TMA), titanium isopropoxide (Ti(OCH(CH_3_)_2_)_4_, TTIP), and water vapor were used as precursors. The chemical compositions of the AlO_x_ and TiO_x_ layers were analyzed by XPS depth profiles, as shown in Supplementary Fig. S3. Subsequently, ITO films in a thickness of 50 nm was deposited in a DC Ar plasma sputtering system at 150 °C. And then, lift-off the PMMA resist to form the AlO_x_/TiO_x_ resistive switching layers and ITO pillar electrode (with the diameter of 80 μm) at the same time. Finally, a Cr (5 nm)/Au (100 nm) film was thermally deposited onto the graphene plane electrode for contact metal, respectively, at a rate of 0.1 Å/s in the vacuum of 4 × 10^−6^ Torr. The contact metal with an area of 100 μm × 100 μm was defined by a shadow mask during the deposition.

### Electrical measurements

Current-voltage characteristics, cycling endurance, and data retention of the devices were measured by a Keithley 4200 semiconductor parameter analyzer. While a DC voltage was applied to the ITO pillar electrode, the graphene plane electrode was grounded. For testing the RRAM device, no forming process was needed to create the primary conducting filaments in the first cycle, but a current compliance was required to avoid the hard breakdown problems.

### Sample preparation for transmission electron microscopy analysis

Specimen of the device at its initial resistance state (IRS) was prepared in a focused ion beam (FIB) system, FEI Helios 600i. TEM and STEM imaging of IRS specimen was conducted in a 200 kV JEOL 2010F transmission electron microscope.

## Electronic supplementary material

Supplementary Information
