# Supplementary material for: Graphene/h-BN Heterostructures for Vertical Architecture of RRAM Design
Source: Sci Rep. 2017 Aug 29;7:9679. doi: 10.1038/s41598-017-08939-2 (PMC5575158; doi:10.1038/s41598-017-08939-2)
Supplement: Supplementary file 1 — Supplementary Information [file 41598_2017_8939_MOESM1_ESM.pdf]

Supplementary Material for Graphene/h-BN Heterostructures for Vertical Architecture of RRAM Design

Yi-Jen Huang[1] and Si-Chen Lee[1]*

[1]*Graduate Institute of Electronics Engineering, National Taiwan University, Taipei, Taiwan*

**E-mail : sclee@ntu.edu.tw*

The optical image of graphene layer (gray color region) and graphene/multilayer h-BN stacked layers (blue color region) in Fig. S1(a) confirms that both of graphene and graphene/multilayer h-BN stacked films exhibit a good quality with very few cracks after they are transferred onto the substrate. The qualities of graphene layer (gray color region) and graphene/multilayer h-BN stacked layers (blue color region) are further analyzed by using a 633 nm He-Ne laser with a confocal focusing Raman system. The Raman spectrum of graphene layer (gray color region) in Fig. S1(b) shows a blue-shift of the G and 2D peaks. The blue-shift of G peak from 1584 cm^{-1} (intrinsic undoped graphene) to 1602 cm^{-1} and 2D peak from 2680 cm^{-1} (intrinsic undoped graphene) to 2695 cm^{-1} indicate that the graphene layer becomes p-doped by the H_2O dopant introduced during the wet transfer process. The D peak (1350 cm^{-1}) is mainly related to the defects in graphene, which may be introduced during the wet transfer process. The Raman spectrum taken in the surface of graphene/multilayer h-BN stacked layers (blue color region) exhibits a characteristic peak at 1366 cm^{-1} that is due to the E_{2g} phonon mode of multilayer h-BN and analogous to the G peak in graphene, as shown in Fig. S1(c).

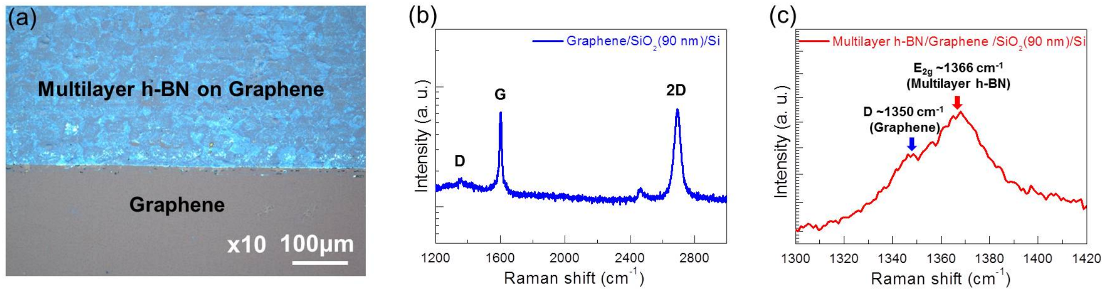

Supplementary Figure S1 | (a) Optical image of graphene layer (gray color region) and graphene/multilayer h-BN stacked layers (blue color region) after the wet transfer

processes. **(b,c)** Raman spectrum taken from the graphene layer (gray color region in (a)) and graphene/multilayer h-BN stacked layers (blue color region in (a)).

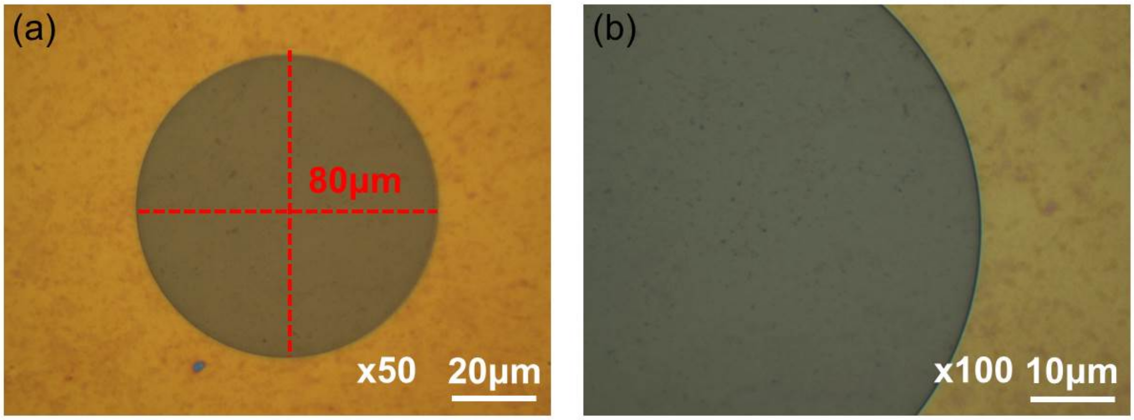

Supplementary Figure S2 | (a,b) Optical images at different magnifications of the vertical hole structures with the diameter of 80 μm after the electron beam lithography (EBL) and dry etching processes, respectively.

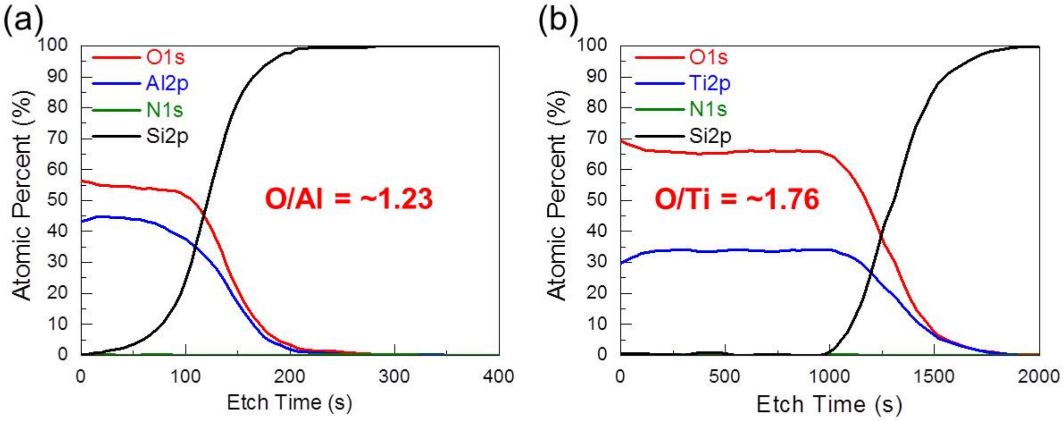

Supplementary Figure S3 | XPS depth profile analysis of **(a)** ALD-AlO_x and (b) ALD-TiO_x films on Si with native oxide substrate. Both the profiles indicate that the chemical compositions are relatively uniform along the film thickness direction. The atomic percent ratio of O/Al and O/Ti are ~1.23 and ~1.76, respectively.
